# 7 Tesla magnetic resonance spectroscopic imaging predicting IDH status and glioma grading

**DOI:** 10.1186/s40644-024-00704-9

**Published:** 2024-05-27

**Authors:** Cornelius Cadrien, Sukrit Sharma, Philipp Lazen, Roxane Licandro, Julia Furtner, Alexandra Lipka, Eva Niess, Lukas Hingerl, Stanislav Motyka, Stephan Gruber, Bernhard Strasser, Barbara Kiesel, Mario Mischkulnig, Matthias Preusser, Thomas Roetzer-Pejrimovsky, Adelheid Wöhrer, Michael Weber, Christian Dorfer, Siegfried Trattnig, Karl Rössler, Wolfgang Bogner, Georg Widhalm, Gilbert Hangel

**Affiliations:** 1 Department of Biomedical Imaging and Image-Guided Therapy, High-Field MR Center, Medical University of Vienna, Vienna, Austria; 2 Department of Neurosurgery, Medical University of Vienna, Währinger Gürtel 18-20, Vienna, A-1090 Austria; 3 A.A. Martinos Center for Biomedical Imaging, Laboratory for Computational Neuroimaging, Massachusetts General Hospital / Harvard Medical School, Charlestown, USA; 4 Department of Biomedical Imaging and Image-Guided Therapy, Computational Imaging Research Lab (CIR), Medical University of Vienna, Vienna, Austria; 5 Division of Neuroradiology and Musculoskeletal Radiology, Department of Biomedical Imaging and Image-Guided Therapy, Medical University of Vienna, Vienna, Austria; 6 Center for Medical Image Analysis and Artificial Intelligence (MIAAI), Danube Private University, Krems, Austria; 7 Division of Oncology, Department of Internal Medicine I, Medical University of Vienna, Vienna, Austria; 8 Division of Neuropathology and Neurochemistry, Department of Neurology, Medical University of Vienna, Vienna, Austria; 9 Institute for Clinical Molecular MRI, Karl Landsteiner Society, St. Pölten, Austria; 10 Christian Doppler Laboratory for MR Imaging Biomarkers, Vienna, Austria; 11 Medical Imaging Cluster, Medical University of Vienna, Vienna, Austria

## Abstract

**Introduction:**

With the application of high-resolution 3D 7 Tesla Magnetic Resonance Spectroscopy Imaging (MRSI) in high-grade gliomas, we previously identified intratumoral metabolic heterogeneities.

In this study, we evaluated the potential of 3D 7 T-MRSI for the preoperative noninvasive classification of glioma grade and isocitrate dehydrogenase (IDH) status. We demonstrated that IDH mutation and glioma grade are detectable by ultra-high field (UHF) MRI. This technique might potentially optimize the perioperative management of glioma patients.

**Methods:**

We prospectively included 36 patients with WHO 2021 grade 2–4 gliomas (20 IDH mutated, 16 IDH wildtype). Our 7 T 3D MRSI sequence provided high-resolution metabolic maps (e.g., choline, creatine, glutamine, and glycine) of these patients’ brains. We employed multivariate random forest and support vector machine models to voxels within a tumor segmentation, for classification of glioma grade and IDH mutation status.

**Results:**

Random forest analysis yielded an area under the curve (AUC) of 0.86 for multivariate IDH classification based on metabolic ratios. We distinguished high- and low-grade tumors by total choline (tCho) / total N-acetyl-aspartate (tNAA) ratio difference, yielding an AUC of 0.99. Tumor categorization based on other measured metabolic ratios provided comparable accuracy.

**Conclusions:**

We successfully classified IDH mutation status and high- versus low-grade gliomas preoperatively based on 7 T MRSI and clinical tumor segmentation. With this approach, we demonstrated imaging based tumor marker predictions at least as accurate as comparable studies, highlighting the potential application of MRSI for pre-operative tumor classifications.

**Supplementary Information:**

The online version contains supplementary material available at 10.1186/s40644-024-00704-9.

## Introduction

Gliomas are the most common primary CNS tumor entities and still challenging for both patients and healthcare providers. Treatment involves resection, post-surgical radiation and chemotherapy, or a combination thereof. Monitoring and management decisions are based on MRI-centered imaging protocols [1, 2]. Pre-operative diagnostics involve contrast-enhanced (CE) MRI to differentiate high- from low-grade gliomas [3–5]. The biopsied samples are classified according to the WHO 2021 guidelines [6], which heavily focus on (epi-)genetic analysis and molecular features to grade and sub-classify gliomas. Tissue-based analyses remain the reference gold standard. Challenges, such as interobserver variability [7] and reliance on invasive surgical biopsy or resection, still remains. In addition to pre-surgical CE imaging [8], MRS provides metabolic information about tumors by mapping oncometabolites such as total choline (tCho) [9], glutamine (Gln), and glycine (Gly) [10]. The presence of an isocitrate dehydrogenase (IDH) mutation might potentially increase glioma cells’ sensitivity to oxidative damage from radiation treatment and molecular targets, e.g., IDH inhibitors [7]. The preoperative precise detection of the IDH mutation, glioma grade and other markers, by ultra-high-field (UHF) MRI potentially benefits the patient by optimizing clinical management.

## Background

Several studies and meta-analyses have investigated MR spectroscopy for glioma classifications. An increase of the MRS markers tCho [11] and 2-hydroxyglutarate (2HG) [12] was found to correlate with IDH mutation. A systematic review and meta-analysis found 2HG-based IDH diagnosis to be 95% sensitive and 91% specific [13]. The current literature proposes 2HG [14], creatine-to-N-acetyl-aspartate (Cr/NAA), and Cho/Cr [11] as most critical for IDH classification. Sampling 1228 patients, Cho, Cr, and NAA based tumor grading revealed a 71–80% sensitivity and a 60–76% specificity [14]. With a novel 7 T spectral-spatial MR spectroscopic imaging (MRSI) technique, we can acquire high-resolution maps of more oncometabolites than previously possible at once [10, 15], providing further data for supervised learning analysis.

The Random Forest (RF) model is useful to explore potential classification features in datasets with previously unknown feature importance weights. Decision trees provide a class prediction and the highest voted class becomes the operating model. Support-Vector Machines (SVM) are supervised machine learning methods that, while avoiding overfitting, operate in both linear and non-linear high-dimensional spaces.

### Purpose

Our 7 T MRSI sequence can map multiple metabolites, including tCho, Gln, Gly, and tNAA at high resolution [10, 15]. We evaluated supervised learning algorithms in segmented metabolic maps for gliomaIDH and grade predictiong.

## Methods

This study was conducted prospectively in accordance with the Declaration of Helsinki and approved by the local institutional review board (number: 1991/2018). For spectroscopic imaging, we included clinically and radiologically suspected low- or high-grade glioma patients prior to the planned surgical resection. Written, informed consent was obtained from all participants. Exclusion criteria were claustrophobia, ferromagnetic implants, non-ferromagnetic metal head implants > 12 mm, pregnancy, and a Karnofsky performance status < 70. The authors of this work had complete control of the study procedures, data analysis, and content of this report. Post-surgical histological diagnosis according to the latest 2021 WHO guidelines [6] provided the gold standard reference for the analysis.

### MRI protocols

We imaged with a concentric ring trajectory-based MRSI sequence on a 7 T Magnetom scanner (Siemens Healthcare, Erlangen, Germany) with a 32-channel receive array coil (Nova Medical, Wilmington, MA, USA), featuring a 64 × 64 × 39 matrix with 3.4 mm^3^ isotropic resolution [15]. The acquisition took 15 min with 450 ms TR and 1.3 ms acquisition delay, covering a manually placed 220 × 220 × 133 mm^3^ field of view (FOV) [10, 15]. More details are found in Supplementary Table 2, which reports MRS parameters in the MRSinMRS standard [16]. We additionally obtained 7 T 0.8 mm3 isotropic T1-weighted MP2RAGE in 8:02 min and 0.8 mm3 isotropic fluid-attenuated inversion recovery (FLAIR) in 8:10 min.

Clinical 3 T MRI consisted of FLAIR, T2-weighted MRI, and pre- and post-contrast T1-weighted MRI (Gadoteridol, 0.1 mmol/kg).

### Post-processing

In-house-developed software postprocessing [15, 17] of MRSI data included gridding, lipid removal by regularization [18], and Hamming filtering. LCModel (v6.3–1, LCMODEL Inc, ONT, CA) spectral fitting included a basis set of N-acetyl-aspartate and NAA-glutamate (tNAA), creatine and phosphocreatine (tCr), tCho, myo-inositol (Ins), scyllo-inositol, γ-aminobutyric acid (GABA), glutathione (GSH), glutamate (Glu), Gln, Gly, taurine (Tau), serine (Ser), cysteine, 2HG and a single macromolecular baseline [17] with an evaluation range of 1.8–4.1 ppm. These formed all the features considered for analysis. A neuro-radiologist with 15 years of specialist experience segmented clinical image-derived tumor regions (i.e., edema or non-contrast enhancing (NCE), CE, and necrosis (NEC)) based on T1, FLAIR and contrast images only, blinded to additional information. We included all spectroscopic voxels within the CE + NCE tumor segmentation that had passed spectral quality filtering [10] (e.g., tCr SNR > 5; tCr FWHM < 0.15 ppm; metabolite Cramér–Rao lower bounds (CRLB) < 40%).

We assessed MRSI quality visually. If most of the tumor focus (i.e. CE and most of NCE) was located in the caudal brain regions with poor spectral coverage, we excluded the whole dataset from further analysis.. Ratio maps of each unique feature denominated by tCr, tCho, and tNAA were established for statistical evaluation and labeled with histologically derived IDH status and tumor grade. We eliminated one in two ratios with a correlation coefficient greater than 0.95. We defined tumor hotspots from which the voxels for the following classifier would be drawn by using lower thresholds based on values obtained from a previous MRSI study in healthy volunteers [19] (i.e., min, mean, and max ratios out of a range of segmented brain ROIs; using three different thresholds to determine how reliant classification was on specific thresholds). Only voxels which were above the threshold for both tCho/tNAA and Gln/tNAA were selected. We eliminated voxels with either tCho/tNAA and Gln/tNAA ratios above 10 as well in order to reduce distortion by very low tNAA fits. Only the remaining of the (all patients) total 55,106 tumour voxels would be used for the RF and SVM.Statistical testing.

We used a Wilcoxon-Mann–Whitney-Test (WMW) to compare key metabolic ratio values of all grade 3 and grade 4 tumor voxels for statistical significant differences in IDHmutation (mt) vs. wildtype (wt).

### Classifier design

Random forest (RF) and SVM-based IDH classification and grading were performed by wrapper-type recursive feature elimination with cross-validation (RFECV) [20] feature selection with area under the curve (AUC) as scoring method. We used an initial set of 33 features, consisting of the tumor voxels’ metabolic ratios (i.e., tNAA, tCr, tCho, Ins, GABA, GSH, Glu, Gln, Gly, Tau, and Ser denominated by (tCr, tCho, and tNAA)). The classification problem was defined as the binomial of each voxels’ IDH and high-grade probability. RF with 10 decision trees, a verbosity of 2, and five-fold cross-validation was used. Training and testing were performed iteratively with leave-one-out cross-validation. In addition, as a reference for comparison, we trained an RF and SVM classifier, based on the in previous studies most discriminably reported single feature tCho/tNAA [8].

The classifier’s prediction probabilities for the labels (IDH or grade) were calculated voxel-wise and then aggregated to arrive at a patient's prediction (e.g., IDH-mt or wt). We explored three different aggregation methods, choosing the dataset's mean (1) and median (2) of IDH-mt and high-grade probabilities. The percentage of a dataset's IDH-positive or high-grade voxel was calculated as the patient's binomial (3) aggregation. RF and SVM prediction was performed over CE + NCE ROIs. For each voxel, a RF and SVM based IDH and HG predictive value was calculated. In each patient dataset, the min/median/mean/max values for these predictions were processed to form the one aggregated patient-based value. Binomial aggregation was an aggregation method, in which each voxels’ predictory IDH and grade values (e.g., 0.2 and 0.7, respectively) were transformed towards binomial representation (in that example, 0 and 1 respectively). There was a negligible impact on the statistical outcomes in binomial versus exact voxel value aggregation (to whole patient value).

## Results

### Data quality

**Fig. 1:**
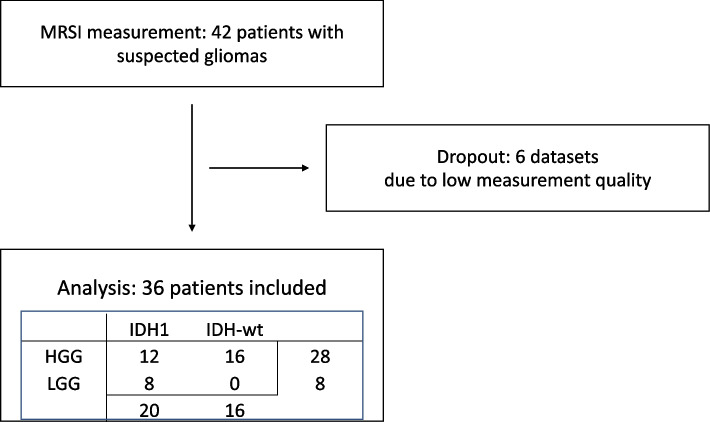
Subject recruitment: We imaged 42 glioma patients. Six datasets were excluded due to low measurement quality due to movement artefacts or tumors located too far caudally for reliable spectroscopic quantification. The final dataset thus included 28 grade 3 and grade 4 tumors (HGG) and eight grade 2 tumors (LGG); 20 with IDH mutation and 16 IDH wildtype

**Fig. 2:**
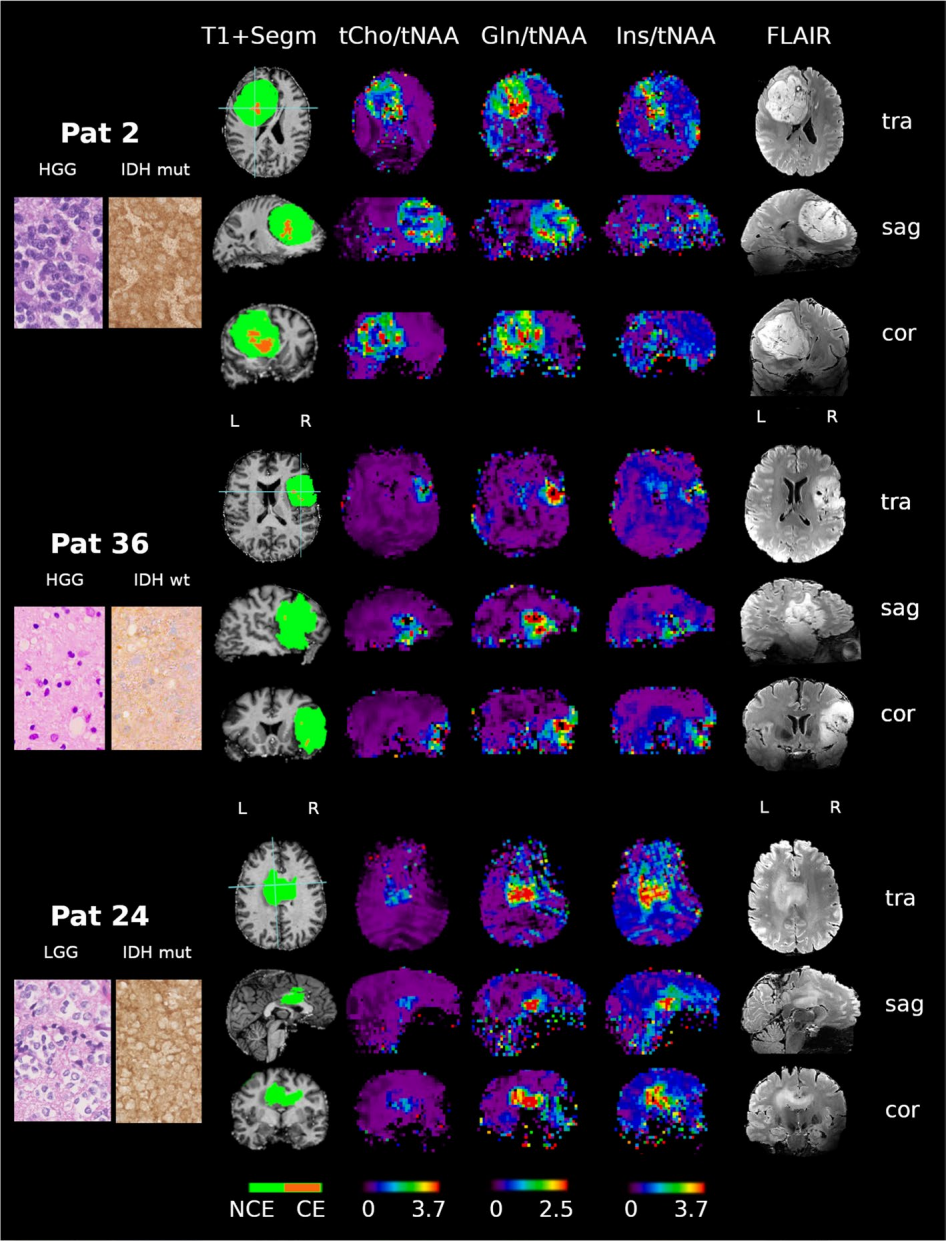
Selected spectroscopic and anatomic maps. Spectroscopic maps are shown in the original resolution of 64 × 64x39 voxels. Segmentation (green = non-contrast-enhancing tumor region; orange = contrast-enhancing tumor region). Note the spectroscopic differences within the segmented tumor regions, especially the differing maximum levels of tCho/tNAA. Astrocytoma 4, IDH-mutant (top); Glioblastoma 4, IDH-wildtype (middle); Oligodendroglioma 2, IDH-mutant (bottom). Metabolic ratios are levelled on the same scale (see the bottom legend): tCho/tNAA: 0–3.7; Gln/tNAA: 0–2.5; Ins/tNAA: 0–3.7. Left column: Histologic slices of the respective tumor with hematoxylin and eosin stain and IDH stain. Note the relatively low stain in IDH-wt Pat 36

We excluded six of 42 datasets (see Fig. 1) for insufficient MRSI quality (movement artefacts or b0-inhomogeneity due to the basal location of the tumor in the brain). In the remaining 36 patients, the whole tumor area was covered by the spectral maps (i.e., parietal lobe). Our study also included 6 patients with a recurrent glioma. Figure 2 shows a graphical overview of some selected datasets. Of the total 55,106 tumor voxels in all patients, yielded elimination of 25–50% of healthy appearing voxels (see Supplementary Table 3). E.g., in the max thresholding scenario, only voxels with tCho/tNAA values between 0.2444 and 10, and Gln/tNAA between 0.2782 and 10, were included for further analysis, thus eliminating 50.2% of the total tumor segmentation voxels. For the mean thresholds (0.166 for tCho/tNAA and 0.199 for Gln/tNAA), this would result in 37,272 voxels or 67.64% remaining for analysis. For minimum ratios, 44,905 or 81.49% remained and for maximum ratios 27,431 voxels or 49.78%.

### Classification

**Fig. 3:**
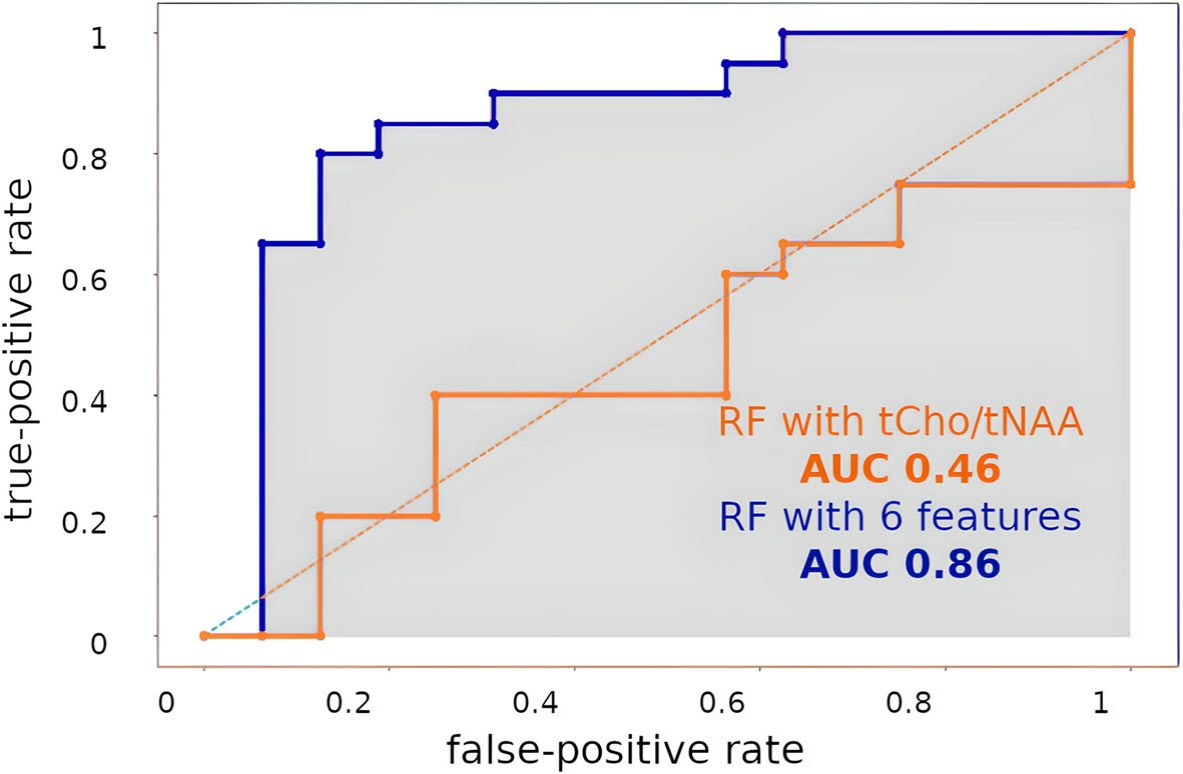
ROC of two selected IDH mutation status classification models. blue: with six features and max. threshold (AUC = 0.86); orange: tCho/tNAA as a single feature and a min. threshold (AUC = 0.46)

Voxel-wise comparison of key metabolic ratios (i.e. tCho, Gln, Glu, Gly, Ins / tNAA, respectively) in IDH-mt vs wt yielded *p* < 0.0001 for differences (see Supplementary Fig. 4). The only exception was a non-significant Glu difference. tCho/tNAA IDH classification resulted in an AUC < 0.45, and multi-feature RF classification yielded an AUC of > 0.84 with more than four features and a mean or max threshold for tumor hotspot selection (see Tables 1 and 2). We identified Glu, Gln, GSH, and Gly as the most crucial for IDH prediction, rated by RF and SVM algorithms with high importance weights in several independent runs (see cross-validation scores in the Supplements). Figure 3 shows the best-performing ROC, compared to tCho/tNAA classification.

**Table 1:**
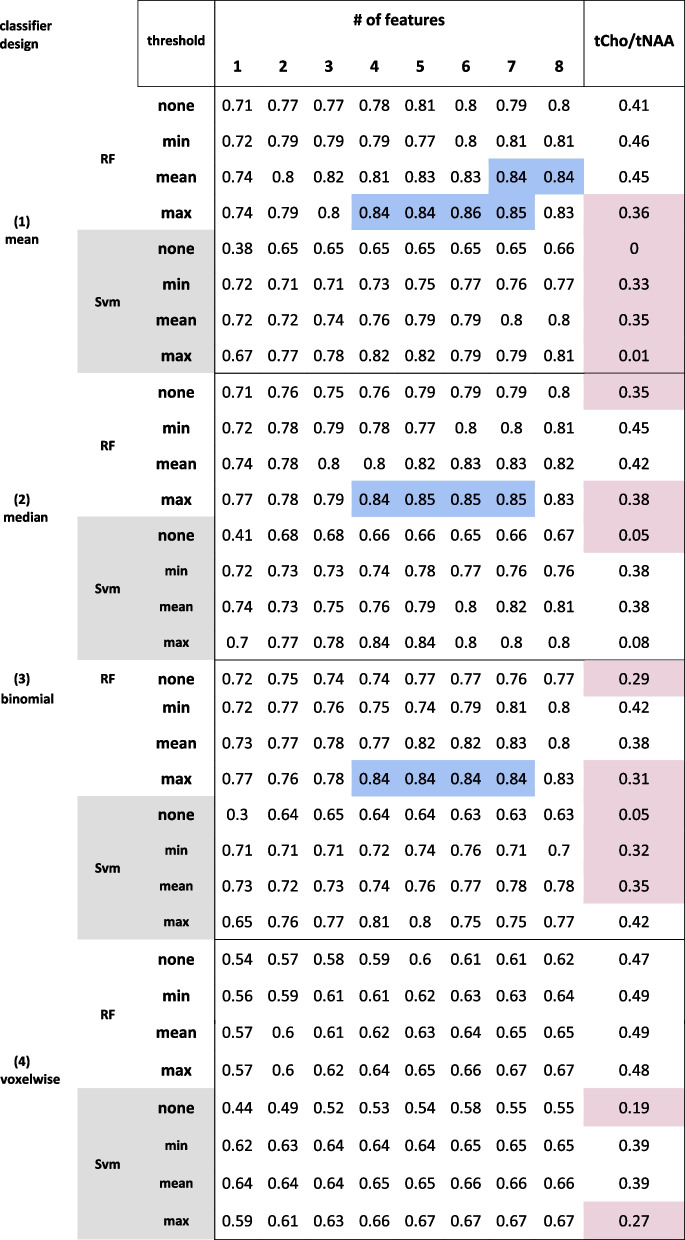
RF and SVM classifiers performance for IDH

Comparison of random forest and SVM IDH classifier results by applied thresholds. Best-performing multi ratio classifier AUC values highlighted in blue; worst performance highlighted in red. For comparison, mean (1), median (2), and binomial (3) probability aggregation methods are shown, along with the raw probabilities of each voxels’ correct classification (4)

**Table 2:**
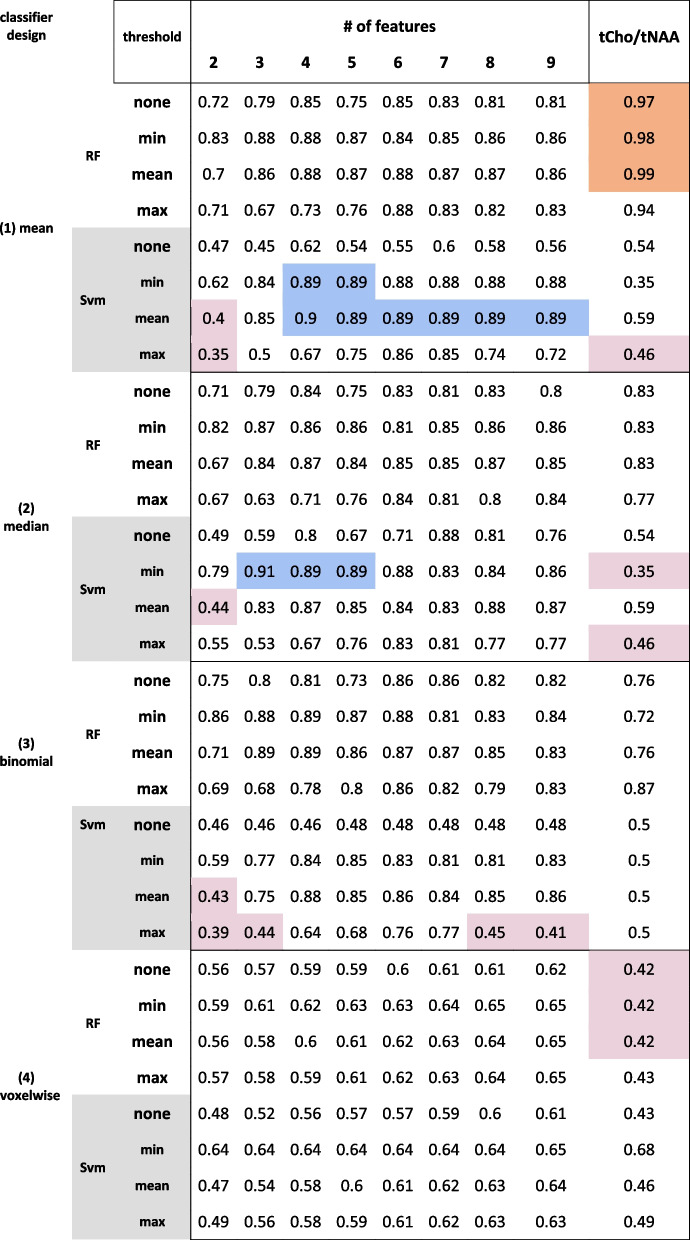
RF and SVM classifiers performance for grade

Comparison of random forest and SVM grade classifier results by applied thresholds. Best-performing single ratio classifier AUC values highlighted in orange; best-performing multi ratio classifier AUC values highlighted in blue; worst performance highlighted in red. For comparison, mean (1), median (2), and binomial (3) probability aggregation methods are shown, along with the raw probabilities of each voxels’ correct classification (4)

**Fig. 4:**
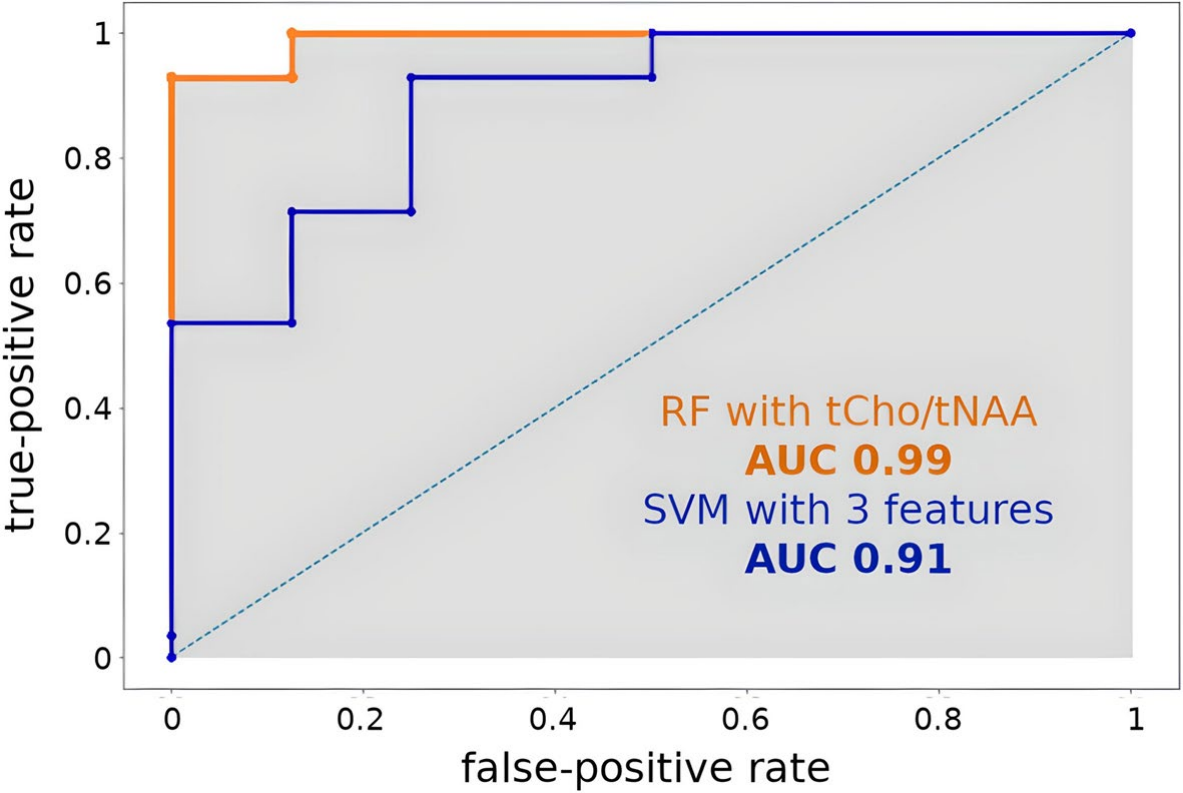
ROC of two selected tumor grade classification models. Blue: with six features and a min. threshold (AUC = 0.91); orange: tCho/tNAA as a feature and a min. threshold (AUC = 0.99)

Tumor-grading yielded an AUC of 0.99 and 0.89 for single feature tCho/tNAA and multi-feature grading, respectively (see Table 1). Ins, Gly, GSH, and Tau appeared to be the key features for grade classification (see CV scores in the Supplements). ROCs of the best-performing classifiers are shown in Fig. 4.

## Discussion

In this study, prediction of IDH mutation status and tumor grade yielded an AUC of 0.86 and 0.99, respectively. Similar single-voxel spectroscopy (SVS) studies at 3 T predicted IDH mutation status with an accuracy of 88% [21, 22]. In comparison, a previous study using MRSI to define different tumor classes was 93–95% accurate [23]. In a meta-analysis, monitoring treatment response, e.g., with IDH inhibitor treatment, provided the highest accuracy with spectroscopic imaging, compared to other MRI techniques [24]. In comparison, most classifier studies use structural imaging methods to discern IDH status and grade. For example, DWI-based [25] IDH classification studies performed with 97% accuracy. While structural imaging may lack direct metabolic information, the availability of more and better standardised datasets has yielded strong results. Other MRS studies [26–30] that involved 2HG and other spectroscopic markers have shown comparable results to our findings. An IDH mutation shifts cell metabolism from aerobic glycolysis to anaerobic glutaminolysis [31], thus altering measurable metabolic profiles.

With a grading AUC of 0.99, we outperformed studies, such as an MR diffusion kurtosis imaging-based meta-analysis that reported an AUC of 0.94 [32]. Because it is a marker of astrocytes, Ins increases in higher tumor grades. According to an European survey, most of the 220 centers use MRS clinically for lesion characterization and tumor-grading [33].

Our results provide an optimistic outlook on the potential of 7 T-3D-MRSI for preoperative tumor-marker prediction. Because of the high resolution, we acquired more tumor voxels for analysis and classification than SVS studies, which heavily rely on a limited number of voxels to arbitrarily encompass the tumor area. Even though we reached accurate IDH and tumour grade predictions, comparable to SVS and other MR-based studies, the potential of 7 T MRSI lies in the high resolution. The broad panel of measurable metabolites enabled us to classify tumors effectively, especially by separating glycine and glutamine. Cross-validation makes our results statistically foundational and reliable. Improved preoperative characterization of gliomas might optimize the perioperative management of glioma patients. For example, the preoperative knowledge of the IDH status would be beneficial to plan a maximal safe tumor resection especially in cases with IDH mutated gliomas [34].

### Limitations

The sample size of 36 limited the statistical power of subgroup analyses and our present study thus focused on the entire glioma cohort. An investigation within distinct entities, such as glioblastomas or IDH mutant astrocytomas, constitutes a worthwhile endeavor for future studies [35]. During this study, we were still relying on manual segmentation, but are currently working on automated segmentation tools. There is still a lack of 7 T scanners in Europe and the US for widespread adoption. Our efforts went towards basing the analysis on tumor voxels with higher SNR, which may discard some high choline and low creatine voxel. In practice, that means that on average there were still > 100 voxel with higher SNR values included per tumor patient, sometimes even thousands. Technical limitations restricted us to the use of metabolite ratios. However, we are working on the implementation of SI-unit-based concentration estimates [19] not only in healthy tissue but also in gliomas. As our free induction decay (FID-)MRSI approach is not sensitive enough for direct 2HG detection, this more straightforward approach for IDH mutation identification is not possible from our data. Adapting a 2HG-tailored acquisition would reduce the speed and resolution of our method. Even though our study yielded insights into tumor classification and metabolism, specific treatments for molecular subtypes must first be approved to make our assets fully contribute to enhancing glioma patient outcomes. We are also working closely with clinicians to employ the technology for surgical delineation.

## Conclusions

We have successfully leveraged 7 T MRSI for glioma classifications. However, this is still an early stage for UHF MRSI in glioma assessment and routine implementation into the clinical workflow would require some further work to address the remaining challenges.

### Outlook

When thinking about an all-encompassing, data-driven diagnostic and treatment approach, UHF spectroscopic imaging can contribute valuable information. In this sense, we might obtain data to classify even more tumor biomarkers non-invasively, and better models may provide more specific information on glioma subtyping (e.g., oligodendroglioma, astrocytoma), aiding patient-level precision medicine and future targeted therapies. In the long term, with enough evidence about MRSI based glioma classifications, improved surgical planning could be performed according to better predictive models about tumor compartments and infiltration. UHF spectroscopic imaging screenings might potentially diagnose incidental brain diseases without clinical symptoms to allow optimal treatment planning at an early stage.

### Supplementary Information

Supplementary Material 1. Supplementary Figure 1: IDH prediction features ranked by their importance. Note that Glu+Gln/tCho, Glu/tCho and GSH/tCho ratios are those with the highest importance scores. IDH prediction with the 6 purple labeled features yielded maximum AUC of 0.86.

Supplementary Material 2. Supplementary Figure 2: RF-based grade prediction features ranked by their importance. Note that Ins/tCho, GSH/tCho and Ins+Gly/tCho ratios are those with the highest importance scores. Grade prediction with the purple labeled features yielded maximum AUC of 0.91.

Supplementary Material 3. Supplementary Figure 3: Sample spectra of Pat 2 and the respective locations within the brain. Normal-appearing white matter (NAWM) shows a distinctively different pattern of metabolic ratios compared to the voxel in the tCho/tNAA hotspot, especially the tCho/tNAA ratio. The presented spectra were not specifically first-order phased, so FID-MRS resonances are out of phase to each other due to phase evolution (at 1.3 ms in our case). The basis set accounted for this phase evolution.

Supplementary Material 4. Supplementary Figure 4: Selected metabolic differences. Boxplots of metabolic differences of all grade 3 and grade 4 tumor voxels with IDH mutation vs. IDH wildtype - compared with a WMW test. **** *p* < 0.0001; ns non significant.

Supplementary Material 5. Supplementary Figure 5: Selected quality maps of Patient 2. Along with the 7T flair and tCho/tCr maps as reference, FWHM, SNR and CRLBs for tCr and other metabolites are plotted. The provided quality maps are clamped to the respective filtering (see bottom scale; see methods section). Notably, even though some CRLB maps show high values throughout the brain, the MRSI seems to have worked well within the tumor area.

Supplementary Material 6. Supplementary Tables.

## Data Availability

The datasets used during this study can be made available by the corresponding author upon reasonable request.

## Abbreviations

2HG: 2-Hydroxyglutarate
AUC: Area under the curve
CE: Contrast-enhanced
tCho: Choline-containing compounds
tCr: Total creatine, creatine + phosphocreatine
CRLB: Cramér–Rao lower bound
FID: Free induction decay
FLAIR: Fluid-attenuated inversion recovery
FOV: Field of view
FWHM: Full width at half maximum
GABA: γ-Aminobutyric acid
Gln: Glutamine
Glu: Glutamate
Gly: Glycine
GSH: Glutathione
HGG: High-grade glioma (includes grade 3 and grade 4 tumors)
IDH: Isocitrate dehydrogenase
Ins: (Myo-)inositol
LGG: (Grade 2 tumor)
MP2RAGE: Magnetization-prepared 2 rapid acquisition gradient echoes
MRSI: Magnetic resonance spectroscopic imaging
Ms: Millisecond(s)
NAA: N-acetyl-aspartate
NAAG: N-acetyl-aspartyl glutamate
NCE: Non-contrast-enhanced
NEC: Necrotic
RF: Random forest
ROI: Region of interest
RFECV: Recursive feature elimination with cross-validation
Ser: Serine
SNR: Signal-to-noise ratio
SVM: Support vector machine
SVS: Magnetic resonance single-voxel spectroscopy
T1w: T1-weighted
T2w: T2-weighted
Tau: Taurine
TR: Repetition time
UHF: Ultra-high-field
WMW: Wilcoxon-Mann–Whitney-Test
Wt: Wildtype
