# Supplementary figures and images for: 7 Tesla magnetic resonance spectroscopic imaging predicting IDH status and glioma grading
Source: Cancer Imaging. 2024 May 27;24:67. doi: 10.1186/s40644-024-00704-9 (PMC11129458; doi:10.1186/s40644-024-00704-9)

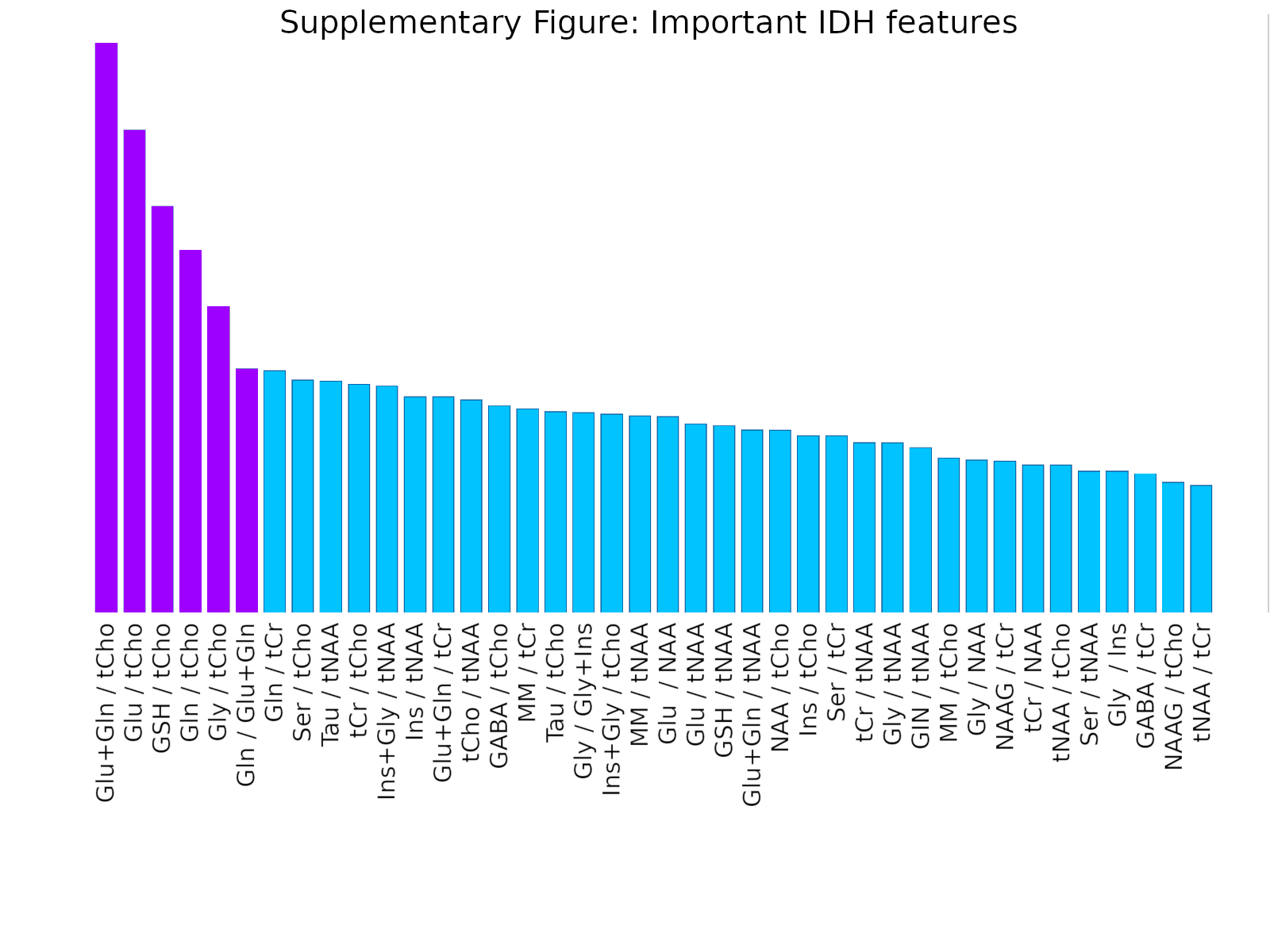

Supplement: Supplementary file 1 — Supplementary Material 1. Supplementary Figure 1: IDH prediction features ranked by their importance. Note that Glu+Gln/tCho, Glu/tCho and GSH/tCho ratios are those with the highest importance scores. IDH prediction with the 6 purple labeled features yielded maximum AUC of 0.86. [file 40644_2024_704_MOESM1_ESM.png]

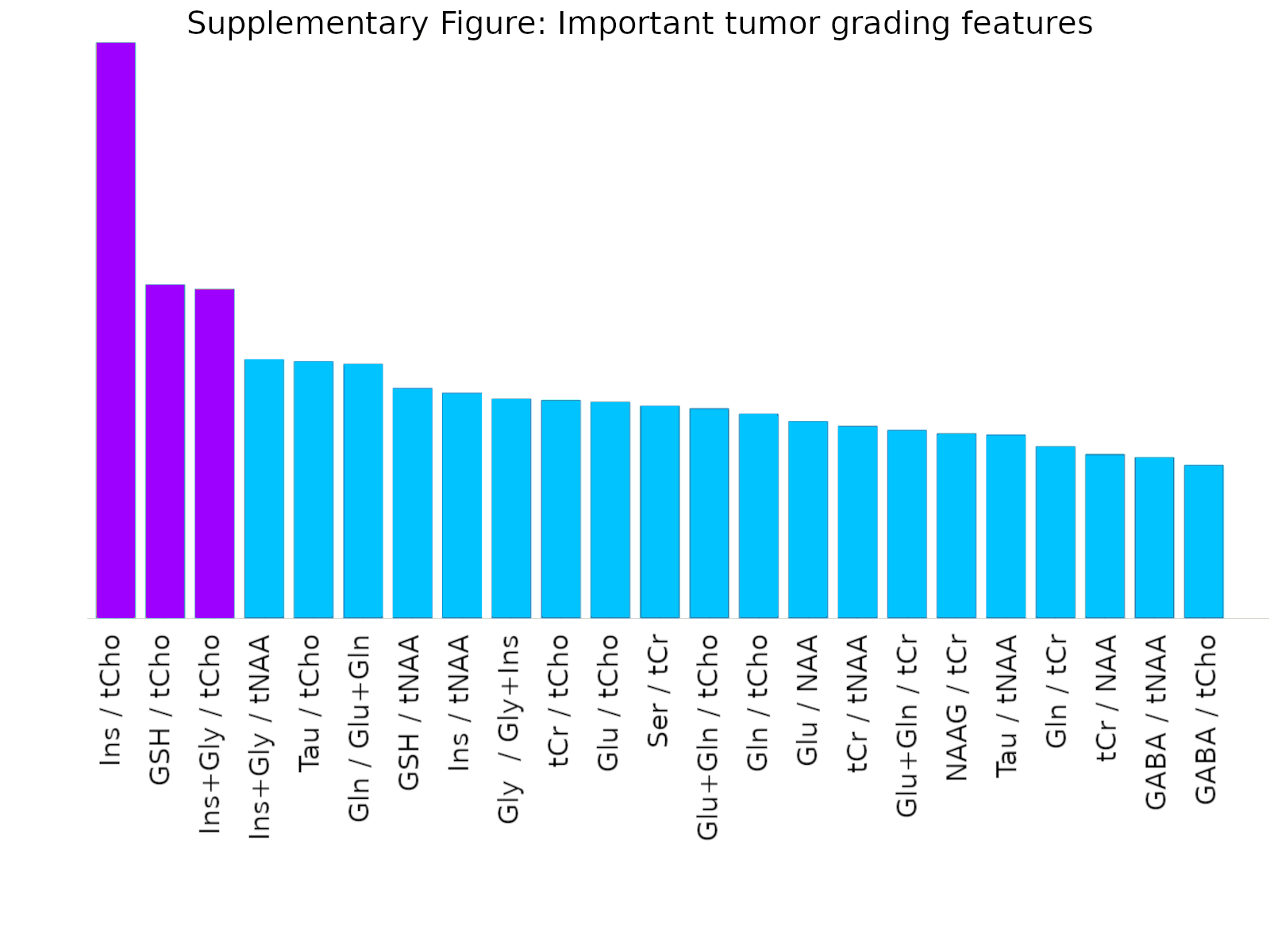

Supplement: Supplementary file 2 — Supplementary Material 2. Supplementary Figure 2: RF-based grade prediction features ranked by their importance. Note that Ins/tCho, GSH/tCho and Ins+Gly/tCho ratios are those with the highest importance scores. Grade prediction with the purple labeled features yielded maximum AUC of 0.91. [file 40644_2024_704_MOESM2_ESM.png]

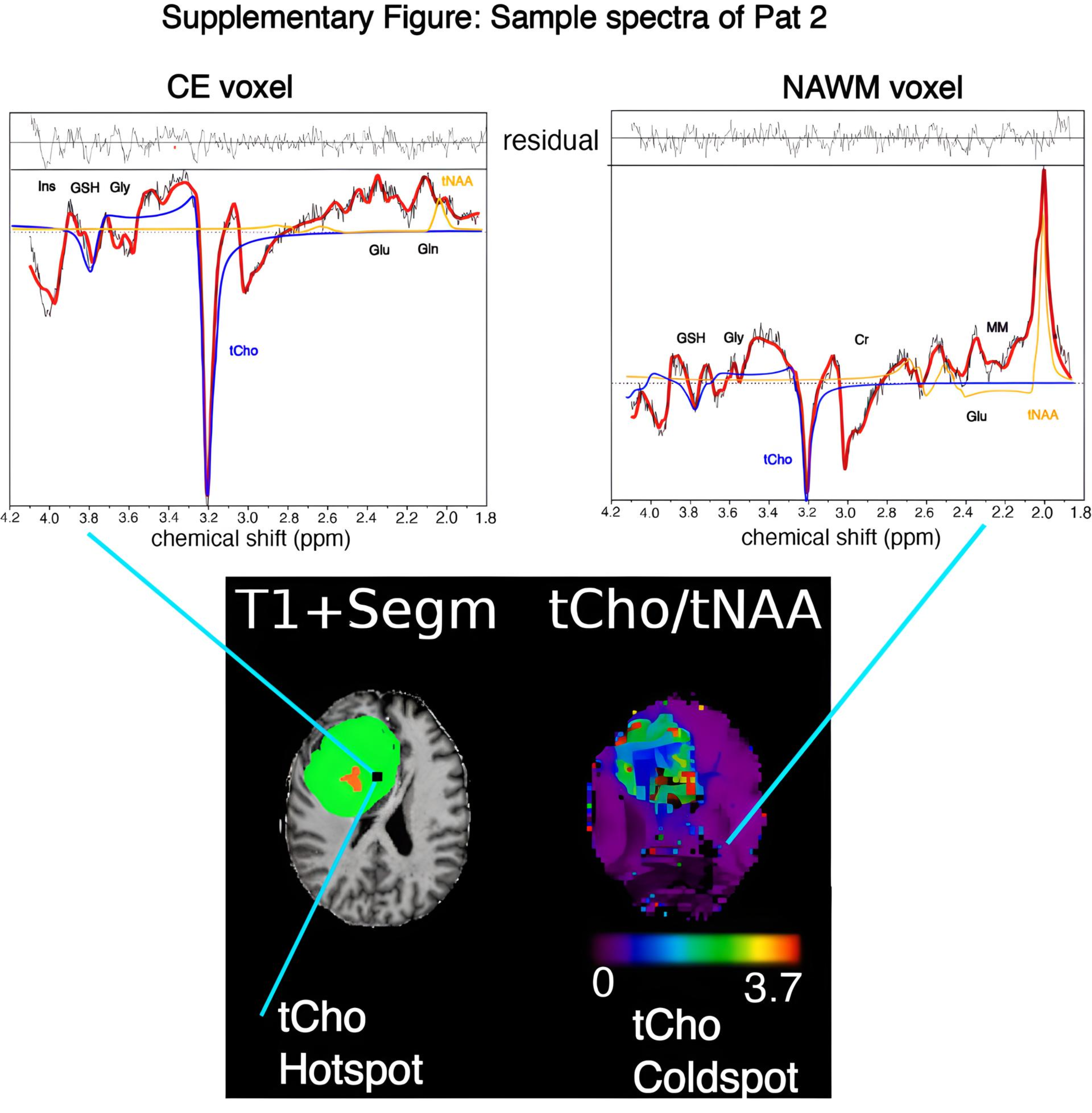

Supplement: Supplementary file 3 — Supplementary Material 3. Supplementary Figure 3: Sample spectra of Pat 2 and the respective locations within the brain. Normal-appearing white matter (NAWM) shows a distinctively different pattern of metabolic ratios compared to the voxel in the tCho/tNAA hotspot, especially the tCho/tNAA ratio. The presented spectra were not specifically first-order phased, so FID-MRS resonances are out of phase to each other due to phase evolution (at 1.3 ms in our case). The basis set accounted for this phase evolution. [file 40644_2024_704_MOESM3_ESM.png]

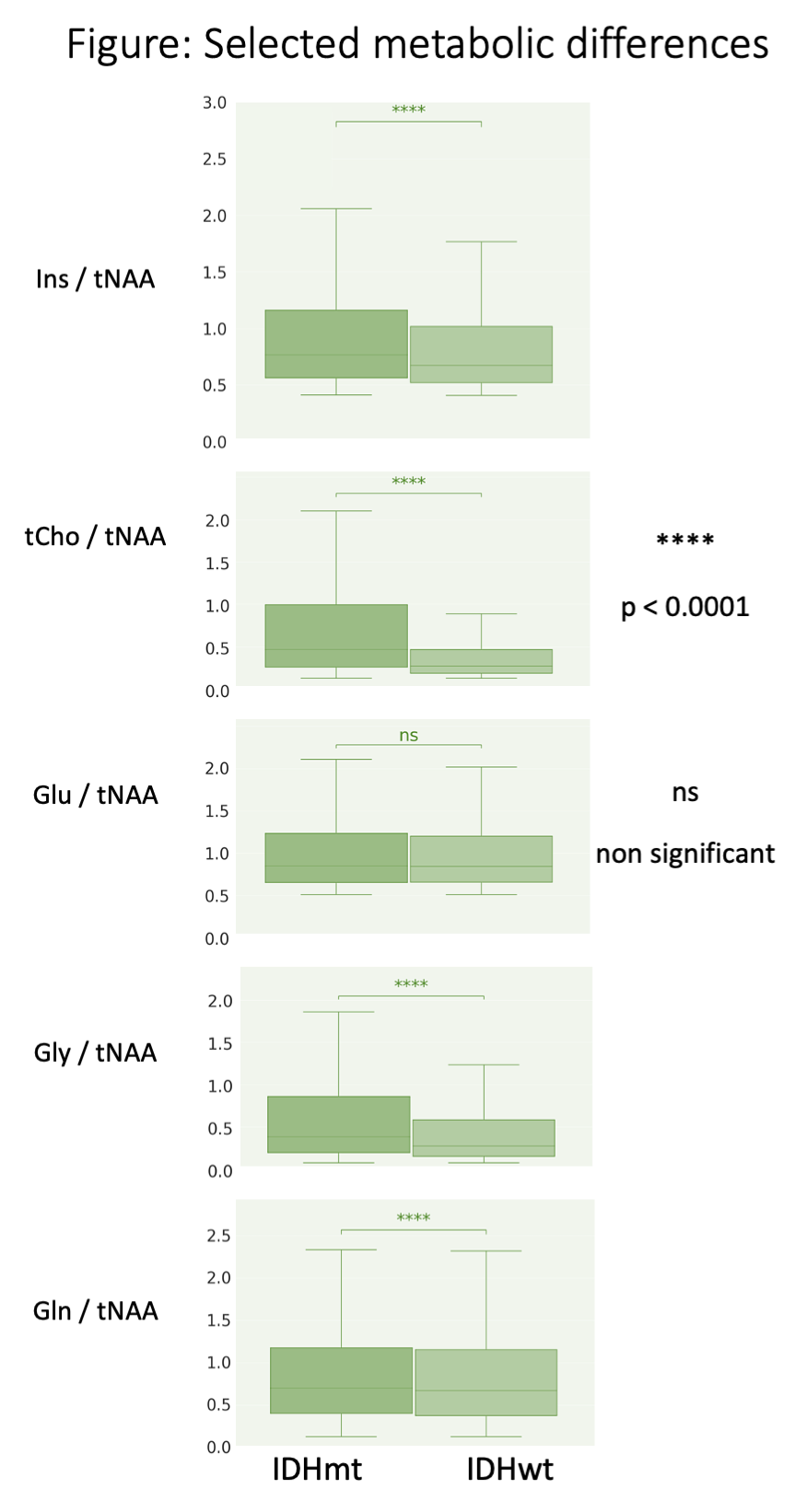

Supplement: Supplementary file 4 — Supplementary Material 4. Supplementary Figure 4: Selected metabolic differences. Boxplots of metabolic differences of all grade 3 and grade 4 tumor voxels with IDH mutation vs. IDH wildtype - compared with a WMW test. **** p < 0.0001; ns non significant. [file 40644_2024_704_MOESM4_ESM.png]

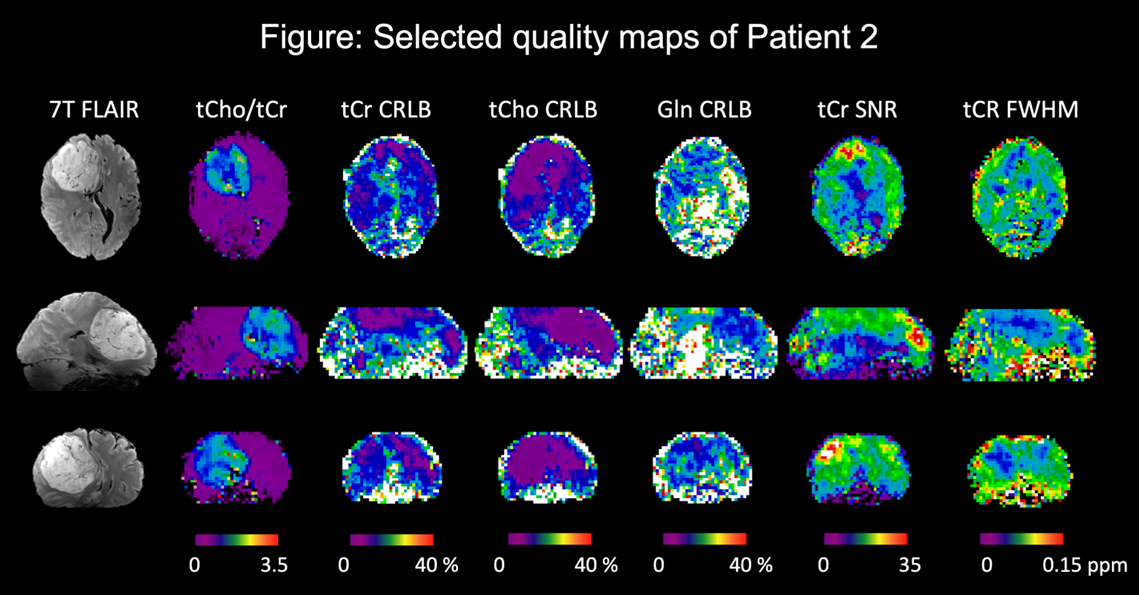

Supplement: Supplementary file 5 — Supplementary Material 5. Supplementary Figure 5: Selected quality maps of Patient 2. Along with the 7T flair and tCho/tCr maps as reference, FWHM, SNR and CRLBs for tCr and other metabolites are plotted. The provided quality maps are clamped to the respective filtering (see bottom scale; see methods section). Notably, even though some CRLB maps show high values throughout the brain, the MRSI seems to have worked well within the tumor area. [file 40644_2024_704_MOESM5_ESM.png]
