# Supplementary material for: 7 Tesla magnetic resonance spectroscopic imaging predicting IDH status and glioma grading
Source: Cancer Imaging. 2024 May 27;24:67. doi: 10.1186/s40644-024-00704-9 (PMC11129458; doi:10.1186/s40644-024-00704-9)
Supplement: Supplementary file 6 — Supplementary Material 6. Supplementary Tables. [file 40644_2024_704_MOESM6_ESM.docx]

# Supplements

### Supplementary Tables

#### Supplementary Table: Histologically confirmed diagnosis

| **Pat. #** | **WHO 2021** | **Grade** | **IDH** | **age** | **sex** |
| --- | --- | --- | --- | --- | --- |
| 1 | Astrocytoma, IDH-mutant | 2 | IDH-mut | 39 | male |
| 2 | Astrocytoma, IDH-mutant | 4 | IDH-mut | 51 | male |
| 3 | Astrocytoma, IDH-mutant | 4 | IDH-mut | 52 | male |
| 4 | Glioblastoma, IDH-wildtype | 4 | wt | 47 | female |
| 5 | Astrocytoma, IDH-mutant | 3 | IDH-mut | 46 | female |
| 6 | Astrocytoma, IDH-mutant | 3 | IDH-mut | 29 | male |
| 7 | Glioblastoma, IDH-wildtype | 4 | wt | 52 | male |
| 8 | Astrocytoma, IDH-mutant | 2 | IDH-mut | 33 | male |
| 9 | Glioblastoma, IDH-wildtype | 4 | wt | 58 | male |
| 10 | Astrocytoma, IDH-mutant | 2 | IDH-mut | 77 | female |
| **11** | **Glioblastoma, IDH-wildtype** | **3** | **wt** | **34** | **female** |
| 12 | Glioblastoma, IDH-wildtype | 4 | wt | 71 | male |
| 13 | Oligodendroglioma, IDH-mut., 1p/19q-codeleted | 3 | IDH-mut | 51 | male |
| 14 | Astrocytoma, IDH-mutant | 3 | IDH-mut | 62 | male |
| 15 | Glioblastoma, IDH-wildtype | 4 | wt | 30 | female |
| 16 | Astrocytoma, IDH-mutant | 2 | IDH-mut | 34 | male |
| 17 | Oligodendroglioma, IDH-mut., 1p/19q-codeleted | 3 | IDH-mut | 56 | male |
| 18 | Glioblastoma, IDH-wildtype | 4 | wt | 61 | male |
| 19 | Glioblastoma, IDH-wildtype | 4 | wt | 60 | male |
| 20 | Astrocytoma, IDH-mutant | 3 | IDH-mut | 28 | female |
| 21 | Glioblastoma, IDH-wildtype | 4 | wt | 62 | male |
| 22 | Oligodendroglioma, IDH-mut., 1p/19q-codeleted | 2 | IDH-mut | 50 | female |
| 23 | Oligodendroglioma, IDH-mut., 1p/19q-codeleted | 2 | IDH-mut | 38 | female |
| 24 | Oligodendroglioma, IDH-mut., 1p/19q-codeleted | 2 | IDH-mut | 61 | male |
| 25 | Glioblastoma, IDH-wildtype | 4 | wt | 77 | female |
| 26 | Astrocytoma, IDH-mutant | 2 | IDH-mut | 33 | male |
| 27 | Glioblastoma, IDH-wildtype | 4 | wt | 75 | female |
| 28 | Glioblastoma, IDH-wildtype | 4 | wt | 58 | male |
| 29 | Oligodendroglioma, IDH-mut., 1p/19q-codeleted | 3 | IDH-mut | 57 | female |
| 30 | Astrocytoma, IDH-mutant | 3 | IDH-mut | 40 | male |
| **31** | **Glioblastoma, IDH-wildtype** | **4** | **wt** | **58** | **male** |
| 32 | Astrocytoma, IDH-mutant | 4 | IDH-mut | 27 | female |
| 33 | Glioblastoma, IDH-wildtype | 4 | wt | 72 | male |
| 34 | Astrocytoma, IDH-mutant | 4 | IDH-mut | 26 | female |
| 35 | Glioblastoma, IDH-wildtype | 4 | wt | 59 | male |
| **36** | **Glioblastoma, IDH-wildtype** | **4** | **wt** | **46** | **female** |
|  |  | **Median** | **+ IQR** | **52 ±**  **23** |  |

Supplementary Table 1: Patient overview. Glioma classifications according to the latest WHO 2021 guidelines. IDH mutation status, age, and sex at time of measurement. Of note, two cases (patients 31 + 36; marked bold in the table) lacking necrosis and microvascular proliferation were classified as glioblastoma due to combined gain on chromosome 7 / loss on chromosome 10 and one case was classified as glioblastoma due to TERT promoter mutation (patient 11, bold).

| Supplementary Table 2 - MRSinMRS checklist | |
| --- | --- |
| Site | Vienna HFMRC |
| 1. Hardware |  |
| a. Field strength [T] | 7 |
| b. Manufacturer | Siemens |
| c. Model (software version if available) | Magnetom 7T |
| d. RF coils: nuclei (transmit/ receive), number of channels, type,  body part | 1H, 32 ch, head, Nova  Medical |
| e. Additional hardware | N/A |
| 2.  Acquisition | |
| a. Pulse sequence | FID-MRSI |
| b. Volume of Interest (VOI) locations | Tumor, NAWM |
| c. Nominal VOI size [cm^3^, mm^3^] | 220×220×110 |
| d. Repetition Time (TR), Echo Time (TE) [ms, s] | 450 ms / 1.3 ms  acquisition delay |
| e. Total number of excitations or acquisitions per spectrum | N/A, spatial-spectral  encoding |
| In time series for kinetic studies | N/A |
| i. Number of averaged spectra (NA) per  time-point | N/A |
| ii. Averaging method (e.g., block-wise or moving  average) | N/A |
| iii. Total number of spectra (acquired / in  time-series) | N/A |
| f. Additional sequence parameters (spectral width in Hz, number of  spectral points, frequency offsets); If STEAM: Mixing Time TM; If MRSI: 2D or  3D, FOV in all directions, matrix size, acceleration factors, sampling method | BW 2778 Hz, 1920  spectral points, MRSI: 3D, 220×220×133 mm³, 64×64×39, spatial-spectral  encoding |
| g. Water Suppression Method | WET |
| h. Shimming method, reference peak, and thresholds for “acceptance  of shim” chosen | Standard shim +  manual adjustment, water peak < 50 Hz |
| i. Triggering or motion correction method (respiratory, peripheral,  cardiac triggering, incl. device used and delays) | N/A |
| 3.  Data analysis methods and outputs | |
| a. Analysis software | LCModel 6.3-1 |
| b. Processing steps deviating from quoted reference or product | N/A |
| c. Output measure (e.g., absolute concentration, institutional  units, ratio) | Ratio (metabolic) |
| d. Quantification references and assumptions, fitting model  assumptions | Simulated in  NMRScope-B, macromolecular background |
| 4.  Data quality | |
| a. Reported variables (SNR, Linewidth (with reference peaks)) | SNR and linewidths  not reported |
| b. Data exclusion criteria | tCr SNR <5; tCr  FWHM >0.15 ppm; metabolite Cramér–Rao lower bounds (CRLB) >40 % |
| c. Quality measures of post-processing model fitting (e.g., CRLB,  goodness of fit, SD of residual) | CRLB |

Supplementary Table 2: MRSinMRS table as required by the MRS report standards consensus

#### Supplementary Table 3: Remaining voxels after cutoff

| **Cutoff Criteria** | **Cutoff Value** | **Voxels Remaining** | **Percentage of Total (%) Voxels Remaining** |
| --- | --- | --- | --- |
| tCho/tNAA (mean) | >0.166, <10 | 42,360 | 76.87 |
| Gln/tNAA (mean) | >0.199, <10 | 37,272 | 67.64 |
| tCho/tNAA (min) | >0.123, <10 | 49,263 | 89.4 |
| Gln/tNAA (min) | >0.128, <10 | 44,905 | 81.49 |
| tCho/tNAA (max) | >0.244, <10 | 31,009 | 56.27 |
| Gln/tNAA (max) | >0.278, <10 | 27,431 | 49.78 |
|  |  |  |  |

Supplementary Table 3: Remaining voxels of the total 55106 tumor voxels in all patients after excluding differently thresholded (mean, min, max) healthy appearing voxels. Specific cutoff values are stated, e.g. in the max thresholding scenario, only voxels with tCho/tNAA values between 0.244 - 10; Gln/tNAA between 0.278 - 10 were included for further analysis

#### Supplementary Table 4: Median measurement quality

| **Pat** | **ROI** | **tCr CRLB** | **tCho CRLB** | **GSH CRLB** | **Gln CRLB** | **Glu CRLB** | **Gly CRLB** | **Ins CRLB** | **tCr FWHM** | **tCr SNR** |
| --- | --- | --- | --- | --- | --- | --- | --- | --- | --- | --- |
|  |  | **(%)** | **(%)** | **(%)** | **(%)** | **(%)** | **(%)** | **(%)** | **(ppm)** |  |
| 1 | NAWM | 8 | 7 | 21 | 31 | 14 | 94 | 11 | 0.06 | 12.55 |
| 1 | Tumor | 7 | 5 | 20 | 16 | 17 | 118 | 8 | 0.07 | 15.62 |
| 2 | NAWM | 7 | 6 | 15 | 18 | 13 | 70 | 10 | 0.07 | 12.48 |
| 2 | Tumor | 8 | 3 | 16 | 10 | 16 | 14 | 15 | 0.06 | 16.57 |
| 3 | NAWM | 5 | 5 | 13 | 15 | 11 | 70 | 8 | 0.06 | 14.39 |
| 3 | Tumor | 7 | 5 | 24 | 12 | 25 | 27 | 21 | 0.05 | 14.28 |
| 4 | NAWM | 7 | 6 | 17 | 12 | 10 | 84 | 8 | 0.07 | 14.43 |
| 4 | Tumor | 6 | 8 | 20 | 6 | 10 | 41 | 18 | 0.05 | 14.59 |
| 5 | NAWM | 5 | 5 | 12 | 19 | 10 | 82 | 7 | 0.05 | 14.22 |
| 5 | Tumor | 5 | 4 | 14 | 8 | 17 | 45 | 8 | 0.05 | 16.11 |
| 6 | NAWM | 5 | 5 | 15 | 21 | 12 | 75 | 9 | 0.06 | 12.52 |
| 6 | Tumor | 7 | 6 | 20 | 18 | 16 | 55 | 12 | 0.06 | 13.19 |
| 7 | NAWM | 5 | 5 | 15 | 21 | 11 | 78 | 7 | 0.06 | 12.78 |
| 7 | Tumor | 10 | 9 | 18 | 9 | 12 | 25 | 29 | 0.07 | 10.9 |
| 8 | NAWM | 5 | 5 | 15 | 25 | 9 | 88 | 7 | 0.05 | 13.19 |
| 8 | Tumor | 7 | 6 | 18 | 15 | 16 | 95 | 8 | 0.06 | 10.64 |
| 9 | NAWM | 5 | 6 | 15 | 25 | 9 | 79 | 8 | 0.06 | 13.15 |
| 9 | Tumor | 8 | 9 | 18 | 15 | 13 | 49 | 13 | 0.08 | 9.34 |
| 10 | NAWM | 9 | 8 | 18 | 24 | 13 | 84 | 11 | 0.07 | 10.99 |
| 10 | Tumor | 10 | 9 | 30 | 22 | 11 | 55 | 9 | 0.07 | 11.25 |
| 11 | NAWM | 5 | 5 | 14 | 10 | 8 | 22 | 6 | 0.06 | 10.98 |
| 11 | Tumor | 5 | 4 | 13 | 7 | 8 | 14 | 7 | 0.06 | 11.27 |
| 12 | NAWM | 5 | 4 | 15 | 17 | 8 | 37 | 6 | 0.06 | 10.48 |
| 12 | Tumor | 6 | 5 | 18 | 9 | 9 | 42 | 11 | 0.06 | 9.26 |
| 13 | NAWM | 5 | 4 | 13 | 17 | 8 | 29 | 6 | 0.06 | 9.48 |
| 13 | Tumor | 6 | 4 | 19 | 11 | 9 | 21 | 8 | 0.05 | 8.76 |
| 14 | NAWM | 6 | 5 | 16 | 23 | 9 | 38 | 7 | 0.06 | 7.11 |
| 14 | Tumor | 9 | 7 | 14 | 10 | 11 | 26 | 10 | 0.08 | 7.24 |
| 15 | NAWM | 4 | 3 | 12 | 14 | 7 | 20 | 5 | 0.05 | 11.57 |
| 15 | Tumor | 4 | 2 | 14 | 9 | 7 | 11 | 5 | 0.05 | 16.89 |
| 16 | NAWM | 5 | 5 | 14 | 20 | 7 | 34 | 6 | 0.05 | 8.53 |
| 16 | Tumor | 7 | 5 | 18 | 11 | 10 | 27 | 7 | 0.06 | 7.44 |
| 17 | NAWM | 6 | 5 | 18 | 17 | 8 | 45 | 7 | 0.06 | 9.04 |
| 17 | Tumor | 6 | 5 | 18 | 15 | 11 | 45 | 9 | 0.06 | 7.66 |
| 18 | NAWM | 6 | 6 | 14 | 14 | 7 | 40 | 7 | 0.06 | 8.35 |
| 18 | Tumor | 9 | 7 | 17 | 11 | 8 | 30 | 12 | 0.08 | 7.63 |
| 19 | NAWM | 5 | 4 | 14 | 20 | 8 | 35 | 7 | 0.05 | 9.05 |
| 19 | Tumor | 6 | 6 | 18 | 8 | 9 | 37 | 13 | 0.06 | 7.55 |
| 20 | NAWM | 4 | 4 | 14 | 16 | 7 | 25 | 7 | 0.05 | 10.63 |
| 20 | Tumor | 6 | 5 | 16 | 10 | 10 | 26 | 9 | 0.08 | 10.19 |
| 21 | NAWM | 5 | 5 | 13 | 20 | 7 | 34 | 6 | 0.06 | 9.55 |
| 21 | Tumor | 7 | 7 | 16 | 13 | 9 | 30 | 9 | 0.08 | 7.23 |
| 22 | NAWM | 6 | 7 | 14 | 15 | 9 | 29 | 8 | 0.07 | 13.04 |
| 22 | Tumor | 5 | 6 | 16 | 9 | 10 | 27 | 8 | 0.06 | 14.93 |
| 23 | NAWM | 5 | 5 | 13 | 15 | 7 | 28 | 6 | 0.05 | 10.57 |
| 23 | Tumor | 4 | 5 | 13 | 11 | 7 | 24 | 5 | 0.04 | 13.16 |
| 24 | NAWM | 5 | 5 | 14 | 21 | 8 | 35 | 7 | 0.07 | 9.06 |
| 24 | Tumor | 6 | 5 | 23 | 10 | 11 | 21 | 10 | 0.07 | 11.82 |
| 25 | NAWM | 7 | 6 | 16 | 13 | 8 | 26 | 8 | 0.06 | 8 |
| 25 | Tumor | 8 | 6 | 17 | 9 | 7 | 25 | 12 | 0.06 | 8.83 |
| 26 | NAWM | 5 | 4 | 13 | 14 | 7 | 29 | 6 | 0.06 | 10.8 |
| 26 | Tumor | 5 | 4 | 12 | 7 | 13 | 18 | 4 | 0.08 | 11.15 |
| 27 | NAWM | 8 | 7 | 14 | 16 | 9 | 30 | 9 | 0.06 | 9.43 |
| 27 | Tumor | 8 | 6 | 13 | 9 | 9 | 12 | 13 | 0.06 | 9.32 |
| 28 | NAWM | 6 | 5 | 17 | 19 | 9 | 40 | 7 | 0.07 | 7.78 |
| 28 | Tumor | 7 | 6 | 17 | 22 | 11 | 47 | 9 | 0.07 | 6.78 |
| 29 | NAWM | 5 | 4 | 14 | 20 | 8 | 36 | 7 | 0.06 | 9.72 |
| 29 | Tumor | 5 | 5 | 16 | 16 | 10 | 33 | 7 | 0.06 | 8.46 |
| 30 | NAWM | 4 | 5 | 10 | 22 | 8 | 37 | 6 | 0.06 | 8.16 |
| 30 | Tumor | 7 | 5 | 15 | 12 | 11 | 19 | 7 | 0.07 | 6.47 |
| 31 | NAWM | 4 | 4 | 10 | 17 | 7 | 38 | 5 | 0.06 | 8.82 |
| 31 | Tumor | 5 | 5 | 11 | 8 | 8 | 27 | 9 | 0.05 | 8 |
| 32 | NAWM | 5 | 5 | 12 | 16 | 8 | 23 | 7 | 0.07 | 8.33 |
| 32 | Tumor | 6 | 4 | 10 | 6 | 11 | 10 | 8 | 0.06 | 9.51 |
| 33 | NAWM | 5 | 6 | 13 | 16 | 9 | 31 | 7 | 0.05 | 9.41 |
| 33 | Tumor | 5 | 3 | 7 | 6 | 7 | 8 | 9 | 0.06 | 12.84 |
| 34 | NAWM | 5 | 5 | 11 | 13 | 8 | 33 | 8 | 0.05 | 8.46 |
| 34 | Tumor | 5 | 4 | 13 | 10 | 12 | 28 | 8 | 0.04 | 9.06 |
| 35 | NAWM | 5 | 5 | 13 | 14 | 8 | 42 | 8 | 0.06 | 9.22 |
| 35 | Tumor | 4 | 4 | 12 | 6 | 7 | 26 | 8 | 0.05 | 12.3 |
| 36 | NAWM | 4 | 4 | 11 | 13 | 9 | 38 | 5 | 0.06 | 9.23 |
| 36 | Tumor | 3 | 4 | 6 | 5 | 9 | 20 | 4 | 0.05 | 16.57 |
| **Median Tumor** | | 6 | 5 | 16 | 10 | 10 | 27 | 9 | 0.06 | 10.42 |
| **Median NAWM** | | 5 | 5 | 14 | 17 | 8 | 37 | 7 | 0.06 | 9.64 |

Supplementary Table 4: Medians of scan quality parameters for tumor and NAWM segmentation per patient. The bottom two rows illustrate the total median values in tumor and NAWM. Note that voxels without an LCM fit for the respective metabolite were excluded from this analysis.
